# Maternal sedentary behavior during pre-pregnancy and early pregnancy and mean offspring birth size: a cohort study

**DOI:** 10.1186/s12884-018-1902-2

**Published:** 2018-06-27

**Authors:** Sylvia E. Badon, Alyson J. Littman, K. C. Gary Chan, Michelle A. Williams, Daniel A. Enquobahrie

**Affiliations:** 1 Department of Epidemiology, University of Washington, Box 357236, Health Sciences Building, 1959 NE Pacific Street, Seattle, WA 98195-7236 USA; 2 Seattle Epidemiologic Research and Information Center, Box 358280, VA Puget Sound, 1660 S Columbian Way, Seattle, WA 98108 USA; 3 Department of Biostatistics, University of Washington, Box 357232, Health Sciences Building, 1705 NE Pacific Street, Seattle, WA 98195-7232 USA; 4 Department of Epidemiology, Harvard T.H. Chan School of Public Health, Kresge Building, 677 Huntington Avenue, Boston, MA 02115 USA; 5 Division of Research, Kaiser Permanente Northern California, 2000 Broadway, Oakland, CA 94612 USA

**Keywords:** Pregnancy, Sedentary behavior, Birthweight, Head circumference, Ponderal index

## Abstract

**Background:**

Sedentary behavior is associated with adverse health outcomes in the general population. Whether sedentary behavior during pregnancy is associated with newborn outcomes, such as birth size, is not established, and previous studies have been inconsistent. While previous research suggests that male and female fetuses respond differently to maternal behaviors, such as physical activity, the role of infant sex in sedentary behavior-birth size associations has not been examined.

**Methods:**

Participants in the Omega study, a cohort in Washington State (1996–2008), reported leisure time sedentary behavior (non-work time spent sitting), light intensity physical activity, and moderate/vigorous leisure time physical activity duration in the year before pregnancy (*N* = 1373) and in early pregnancy (*N* = 1535, mean 15 weeks). Offspring birth size was abstracted from delivery records. Non-parametric calibration weighting was used to assign adjustment weight (matching the distribution of sociodemographic and medical characteristics of the full cohort (*N* = 4128)) to participants with available sedentary behavior data. Weighted linear regression models were used to estimate mean differences in offspring birthweight, head circumference, and ponderal index (birthweight/length^3^) associated with leisure time sedentary behavior. Regression models were run overall and stratified by offspring sex. Isotemporal substitution modeling was used to determine mean differences in birthweight associated with replacing sedentary behavior with light or moderate/vigorous physical activity.

**Results:**

On average, women spent 2.3 and 2.6 h/day in leisure time sedentary behavior during pre- and early pregnancy, respectively. There were no associations of pre-pregnancy leisure time sedentary behavior with mean birthweight, head circumference, or ponderal index (adjusted β = − 12, 95% CI: -28, 4.1; β = 0.0, 95% CI: -0.04, 0.1; and β = 0.1, 95% CI: -0.2, 0.4, respectively). Early pregnancy sedentary behavior was not associated with mean birth size. Associations of sedentary behavior with mean birth size did not differ by offspring sex. Replacing sedentary time with light or moderate/vigorous physical activity was not associated with mean birthweight.

**Conclusions:**

We did not observe associations of maternal sedentary behavior during pre- or early pregnancy with mean offspring birth size. Pre-pregnancy and early pregnancy sedentary behavior may have important adverse effects on maternal health, but our results do not support associations with mean offspring birth size.

## Background

A growing body of literature has identified prolonged sedentary behavior (low energy expenditure waking behavior while in a sitting, reclining, or lying posture [1]), independent of moderate to vigorous leisure time physical activity, as a risk factor for mortality, diabetes mellitus, and cardiovascular disease [2, 3]. Recognizing the adverse health impact of sedentary behavior, the American Heart Association recently issued an advisory to “Sit less, move more” [4], and the American College of Sports Medicine issued recommendations for reduction of total time spent in sedentary behavior in addition to regular physical activity [5].

Similar to the general population, pregnant women may spend as much as half of their day being sedentary [6, 7]. Sedentary behavior during pregnancy has been associated with increased risk for gestational diabetes mellitus [8], a strong risk factor for macrosomia [9]. The adverse cardiometabolic changes associated with maternal sedentary behavior, including increased blood pressure and triglycerides in addition to changes in glucose metabolism [10], may affect the intrauterine environment and fetal development. An altered intrauterine environment may adversely affect fetal nutrition in the short term, possibly resulting in increased risk for fetal overgrowth, and programming of somatotrophic axes regulating metabolism and postnatal growth in the long term [11].

Previous reports of associations of maternal sedentary behavior during pregnancy and offspring birth size, an indicator of newborn health and risk of future disease, have been inconsistent. Both low and high birthweight and high ponderal index are associated with greater risk of obesity and cardiovascular disease in later life [12–14]. Smaller head circumference is associated with impaired neurological development and lower intelligence in childhood [15]. Several studies have observed associations of sedentary behavior during pregnancy and lower birthweight [16, 17], while another study has observed increased risk of offspring macrosomia [18]; however, other studies have found no associations [19–21]. All previous studies have considered sedentary behavior independently from other leisure time activities, such as light intensity or moderate/vigorous intensity physical activity, which sedentary behavior may be replacing. Leisure time sedentary behavior, light intensity physical activity, and moderate/vigorous physical activity can be considered together using substitution modeling as a more informative approach to the relationship between leisure time behaviors. This approach considers substitution of one type of activity for another, rather than independent associations of each type of activity in single activity regression models. Moreover, despite sex-specific fetal growth patterns [15] and sex-specific differences in response to changes in the intrauterine environment [16] and maternal behaviors, such as physical activity [17], the role of infant sex in associations of sedentary behavior and offspring birth size has not been examined.

The objective of this study was to investigate associations of maternal pre-pregnancy and early pregnancy leisure time sedentary behavior (measured using TV watching and quiet activities done while sitting) with offspring birth size using single activity regression models and substitution modeling, and to examine if associations differ by offspring sex.

## Methods

### Study setting and study population

Data from the Omega study, a prospective pregnancy cohort, were used for this analysis. Details about the study design and data collection have been published previously [22]. Briefly, pregnant women were recruited from clinics associated with Swedish Medical Center and Tacoma General Hospital in Washington State from 1996 to 2008. Women were eligible to participate in the Omega study if they were at least 18 years old, were able to speak and read English, initiated prenatal care prior to 20 weeks gestation, and planned to carry the pregnancy to term and deliver at one of the two study hospitals. Of 5063 eligible women who were approached, 4602 agreed to participate (91%). The Omega study was approved by the Institutional Review Boards of Swedish Medical Center and Tacoma General Hospital. All participants gave written informed consent.

Sedentary behavior data are limited to participants who enrolled in the Omega study between 2003 and 2008, as information on sedentary behavior was only collected during this period of the study (*N* = 1484 for pre-pregnancy, *N* = 1658 for early pregnancy). Participants with sedentary behavior data, live singleton births, and available data on birthweight were included in the current analyses (*N* = 1406 for pre-pregnancy, *N* = 1568 for early pregnancy). Participants with missing data for smoking (*N* = 11), alcohol use (N = 16), or other covariates (*N* = 6) were excluded. A total of 1373 study participants remained for pre-pregnancy analyses and 1535 remained for early pregnancy analyses after these exclusions (Table 1).

**Table 1:** Maternal and offspring characteristics of the full Omega cohort and participants included in the current pre-pregnancy and early pregnancy analyses

	Full Omega cohort (*n* = 4128)	Pre-pregnancy analysis (*n* = 1373)	Early pregnancy analysis (*n* = 1535)
Maternal characteristics			
Age (years), *mean (SD)*	33 (5)	33 (4)	33 (4)
Non-Hispanic white race, *n (%)*	3504 (85)	1196 (87)	1329 (87)
High school education or more, *n (%)*	3733 (96)	1337 (97)	1496 (97)
Married, *n (%)*	3746 (91)	1263 (92)	1417 (92)
Nulliparous, *n (%)*	2510 (61)	803 (58)	894 (58)
Pre-pregnancy BMI, *n (%)*			
Underweight (< 18.5 kg/m^2^)	76 (2)	28 (2)	31 (2)
Normal weight (18.5–24.9 kg/m^2^)	2961 (72)	997 (73)	1121 (73)
Overweight (25–29.9 kg/m^2^)	700 (17)	250 (18)	273 (18)
Obese (≥30 kg/m^2^)	391 (9)	98 (7)	110 (7)
Total gestational weight gain (kg), *mean (SD)*	14 (5)	14 (4)	14 (5)
Maternal behaviors			
Smoking during pregnancy, *n (%)*	225 (6)	71 (5)	80 (5)
Alcohol use during pregnancy, *n (%)*	375 (9)	167 (12)	172 (11)
Pre-pregnancy sedentary time (hours per day), *mean (SD)*	2.3 (1.4)	2.3 (1.3)	2.3 (1.3)
Early pregnancy sedentary time (hours per day), *mean (SD)*	2.6 (1.6)	2.7 (1.6)	2.6 (1.5)
Pre-pregnancy moderate/vigorous leisure time physical activity (hours per week), *median (IQR)*	4.3 (5.4)	4.5 (4.7)	4.4 (4.6)
Early pregnancy moderate/vigorous leisure time physical activity (hours per week), *median (IQR)*	3.0 (5.7)	2.3 (3.6)	2.3 (3.8)
Offspring characteristics			
Birthweight (grams), *mean (SD)*	3449 (555)	3447 (524)	3440 (534)
Gestational age at delivery (weeks), *mean (SD)*	39 (2)	39 (2)	39 (2)
Male, *n (%)*	2110 (51)	738 (54)	830 (54)

### Data collection

Study participants completed an in-person structured interview with a trained study interviewer at an average of 15 weeks gestation. Information collected during the interview included sociodemographic characteristics (maternal age, race, marital status), reproductive and medical history (parity, height, pre-pregnancy weight), and behaviors before and during pregnancy (sedentary behavior, physical activity, smoking and alcohol use). Participants were followed until delivery, and study personnel abstracted medical records for information on course and outcomes of the pregnancy (birth size, gestational age at delivery, and offspring sex). Pre-pregnancy body mass index (BMI) was calculated using reported height and pre-pregnancy weight and categorized according to standard cutoffs (underweight: < 18.5 kg/m^2^, normal: 18.5–24.9 kg/m^2^, overweight: 25–29.9 kg/m^2^, obese: ≥30 kg/m^2^).

### Sedentary behavior

Pre-pregnancy leisure time sedentary behavior was assessed using the following questions: 1) “In the year before you became pregnant, how many hours per day did you sit quietly and watch TV?” and 2) “In the year before you became pregnant, how many hours per day did you sit quietly and perform an activity such as reading or knitting?”. Early pregnancy leisure time sedentary behavior was assessed using the same questions but for the period of time since becoming pregnant. Total leisure time sedentary behavior per day was calculated by summing responses for these two questions, separately for pre-pregnancy and early pregnancy. Participants were also categorized into groups based on quartiles of pre-pregnancy sedentary behavior as well as quartiles of early pregnancy sedentary behavior.

### Birth size

Birthweight (g), head circumference (cm), and birth length (cm) were abstracted from infant medical records. Measurements were made immediately after birth and recorded to the nearest 0.5 cm and 1 g by hospital personnel. Ponderal index (kg/m^3^) was calculated using birthweight and birth length (birthweight/birth length^3^).

### Statistical analysis

Maternal characteristics, maternal behaviors, and offspring characteristics were summarized for the full Omega cohort, the pre-pregnancy analytic population, and the early pregnancy analytic population. Continuous variables were described using mean and standard deviation. Categorical variables were described using frequency and percentage.

Participants with available data for pre-pregnancy or early pregnancy sedentary behavior were assigned adjustment weights (to match the distribution of age, race, parity, pre-pregnancy BMI, and marital status of participants with live, singleton births and available birthweight data in the full Omega study cohort, *N* = 4128) using non-parametric calibration weighting [23]. This missing data approach increases the precision of estimates over a complete case analysis because it uses information from the full study cohort [24]. Weighted linear regression was used to estimate mean differences and 95% confidence intervals (CI) for birthweight, head circumference, and ponderal index for maternal sedentary behavior as a continuous and categorical (quartiles) exposure. Models were run separately for pre-pregnancy and early pregnancy. Sedentary behavior quartiles were also modeled as a continuous variable to determine *P* values for linear trend. Regression models were adjusted for maternal age, race (white/non-white), nulliparity (Y/N), at least high school education (Y/N), married (Y/N), pre-pregnancy BMI category (underweight/normal weight/overweight/obese), smoking during pregnancy (Y/N), alcohol use during pregnancy (Y/N), moderate to vigorous leisure time physical activity (quartiles) in pre-pregnancy or early pregnancy (depending on exposure), gestational age at delivery (weeks), and offspring sex. Models were also run stratified by offspring sex. Two-way multiplicative interaction terms and corresponding *P* values were used to assess interaction by offspring sex.

We also conducted isotemporal substitution modeling analyses [25] to determine mean differences in offspring birthweight associated with replacing leisure time sedentary behavior with light physical activity or moderate/vigorous physical activity. For these analyses, light physical activity duration was calculated from reported activities with a metabolic equivalent of task (MET) value < 3 [26]. The models contain terms for total leisure time duration (calculated as the sum of average leisure time moderate/vigorous physical activity, light physical activity, and sedentary behavior), moderate/vigorous leisure time physical activity duration, and light leisure time physical activity duration, and covariates listed previously. Models were run separately for pre-pregnancy and early pregnancy. Results from this model are interpreted as the mean change in birthweight of increasing one parameter (hours per week of light or moderate/vigorous physical activity) at the expense of time spent in sedentary behavior (the component of total leisure time not included as a variable in the model), while keeping the other variables constant. Isotemporal substitution models were also stratified by low/high sedentary time. High sedentary time was defined using the median sedentary time (14 h/week in pre-pregnancy, 17 h/week in early pregnancy). Two way multiplicative interaction terms and corresponding *P* values were used to assess interaction by low/high sedentary time.

A two-sided alpha level of 0.05 was used for statistical significance in all analyses. Analyses were performed using SAS 9.4 (SAS Institute Inc., Cary NC) and R 3.0.2 [27].

## Results

Omega participants were 33 years old, on average. The majority were non-Hispanic white, married, and 97% had more than a high school education (Table 1). Women spent about 2 h per day in sedentary time pre-pregnancy and about 3 h in sedentary time during early pregnancy. Pre-pregnancy and early pregnancy sedentary time were strongly correlated (ρ = 0.6). Characteristics were similar for participants included in pre-pregnancy or early pregnancy analyses and participants in the full Omega study.

Although mean offspring birthweight was lower by 12 g (95% CI: -28, 4.1) for each additional hour spent in pre-pregnancy sedentary behavior, there were no statistically significant associations between pre-pregnancy leisure time sedentary behavior or early pregnancy leisure time sedentary behavior and mean offspring birthweight (Table 2). There were no associations of pre-pregnancy or early pregnancy leisure time sedentary behavior with mean head circumference or ponderal index. Associations of pre-pregnancy or early pregnancy sedentary behavior with mean birth size were similar in male and female offspring (Table 3).

**Table 2:** Associations of pre-pregnancy and early pregnancy sedentary time with offspring birth size

	N	Mean difference^a^ (95% CI)		N	Mean difference^a^ (95% CI)
*Birthweight (g)*					
Pre-pregnancy sedentary time (hours/day)			Early pregnancy sedentary time (hours/day)		
Continuous (hrs)	1373	−12 (−28, 4.1)	Continuous (hrs)	1535	−0.8 (−15, 13)
Quartile 1 (0.0–1.4)	327	Reference	Quartile 1 (0.0–1.5)	418	Reference
Quartile 2 (1.5–2.0)	409	−29 (−90, 31)	Quartile 2 (1.6–2.4)	340	11 (−48, 69)
Quartile 3 (2.1–2.9)	220	−46 (− 119, 26)	Quartile 3 (2.5–3.1)	395	16 (−42, 75)
Quartile 4 (3.0–13)	417	−51 (− 114, 11)	Quartile 4 (3.2–13)	382	−18 (−80, 44)
P for trend		0.11	P for trend		0.64
*Head circumference (cm)*					
Pre-pregnancy sedentary time (hours/day)			Early pregnancy sedentary time (hours/day)		
Continuous (hrs)	1362	0.0 (−0.04, 0.1)	Continuous (hrs)	1524	0.1 (−0.03, 0.2)
Quartile 1 (0.0–1.4)	323	Reference	Quartile 1 (0.0–1.5)	413	Reference
Quartile 2 (1.5–2.0)	405	0.2 (−0.1, 0.6)	Quartile 2 (1.6–2.4)	338	−0.2 (− 0.5, 0.1)
Quartile 3 (2.1–2.9)	220	0.6 (0.2, 0.9)	Quartile 3 (2.5–3.1)	393	0.1 (−0.2, 0.4)
Quartile 4 (3.0–13)	414	0.1 (−0.2, 0.4)	Quartile 4 (3.2–13)	380	0.1 (−0.3, 0.5)
P for trend		0.45	P for trend		0.40
*Ponderal index (kg/m* ^*3*^ *)* ^b^					
Pre-pregnancy sedentary time (hours/day)			Early pregnancy sedentary time (hours/day)		
Continuous (hrs)	1364	0.1 (− 0.2, 0.4)	Continuous (hrs)	1525	0.0 (− 0.3, 0.4)
Quartile 1 (0.0–1.4)	324	Reference	Quartile 1 (0.0–1.5)	414	Reference
Quartile 2 (1.5–2.0)	406	0.6 (−0.5, 1.6)	Quartile 2 (1.6–2.4)	339	−1.5 (−3.1, 0.2)
Quartile 3 (2.1–2.9)	219	0.5 (−0.7, 1.7)	Quartile 3 (2.5–3.1)	392	−0.8 (−2.5, 0.8)
Quartile 4 (3.0–13)	415	0.6 (−0.2, 1.5)	Quartile 4 (3.2–13)	380	−0.7 (− 2.5, 1.0)
P for trend		0.25	P for trend		0.53

^a^Model is adjusted for maternal age (years), white race, nulliparity, pre-pregnancy BMI category (underweight/normal weight/overweight/obese), high school education or more, marital status (married/single), smoking during pregnancy, alcohol use during pregnancy, gestational age at delivery (weeks), offspring sex, and pre-pregnancy moderate/vigorous leisure time physical activity duration (quartiles) for pre-pregnancy sedentary time analyses or early pregnancy moderate/vigorous leisure time physical activity duration (quartiles) for early pregnancy sedentary time analyses

^b^Results are excluding outliers for ponderal index (> 400 kg/m^3^)

**Table 3:** Associations of pre-pregnancy and early pregnancy sedentary time with offspring birth size by offspring sex

Females			Males		
	N	Mean difference^a^ (95% CI)		N	Mean difference^a^ (95% CI)
*Birthweight (g)*					
Pre-pregnancy sedentary time (hours/day)			Pre-pregnancy sedentary time (hours/day)		
Continuous (hrs)	635	−9.9 (− 29, 9.6)	Continuous (hrs)	738	−14 (− 41, 13)
Quartile 1 (0.0–1.4)	139	Reference	Quartile 1 (0.0–1.4)	188	Reference
Quartile 2 (1.5–2.0)	186	−6.8 (− 96, 84)	Quartile 2 (1.5–2.0)	223	− 39 (− 120, 43)
Quartile 3 (2.1–2.9)	105	−68 (− 175, 39)	Quartile 3 (2.1–2.9)	115	−14 (− 110, 83)
Quartile 4 (3.0–13)	205	−54 (− 145, 37)	Quartile 4 (3.0–13)	212	−38 (− 124, 48)
P for trend		0.15	P for trend		0.52
P for interaction		continuous = 0.37, quartiles = 0.70			
Early pregnancy sedentary time (hours/day)			Early pregnancy sedentary time (hours/day)		
Continuous (hrs)	705	1.8 (−17, 21)	Continuous (hrs)	830	−1.1 (−22, 20)
Quartile 1 (0.0–1.5)	190	Reference	Quartile 1 (0.0–1.5)	228	Reference
Quartile 2 (1.6–2.4)	145	44 (−46, 135)	Quartile 2 (1.6–2.4)	195	−14 (− 88, 61)
Quartile 3 (2.5–3.1)	195	44 (−44, 132)	Quartile 3 (2.5–3.1)	200	5.7 (−70, 81)
Quartile 4 (3.2–13)	175	−4.4 (− 96, 88)	Quartile 4 (3.2–13)	207	− 17 (− 102, 68)
P for trend		0.97	P for trend		0.81
P for interaction		continuous = 0.45, quartiles = 0.71			
*Head circumference (cm)*					
Pre-pregnancy sedentary time (hours/day)			Pre-pregnancy sedentary time (hours/day)		
Continuous (hrs)	631	0.0 (−0.04, 0.1)	Continuous (hrs)	731	0.1 (− 0.1, 0.2)
Quartile 1 (0.0–1.4)	138	Reference	Quartile 1 (0.0–1.4)	185	Reference
Quartile 2 (1.5–2.0)	184	0.5 (0.02, 0.9)	Quartile 2 (1.5–2.0)	221	0.1 (−0.4, 0.5)
Quartile 3 (2.1–2.9)	105	0.7 (0.3, 1.2)	Quartile 3 (2.1–2.9)	115	0.5 (− 0.1, 1.1)
Quartile 4 (3.0–13)	204	0.1 (−0.3, 0.4)	Quartile 4 (3.0–13)	210	0.2 (−0.3, 0.7)
P for trend		0.99	P for trend		0.29
P for interaction		continuous = 0.84, quartiles = 0.51			
Early pregnancy sedentary time (hours/day)			Early pregnancy sedentary time (hours/day)		
Continuous (hrs)	701	0.1 (−0.1, 0.2)	Continuous (hrs)	823	0.0 (−0.1, 0.2)
Quartile 1 (0.0–1.5)	189	Reference	Quartile 1 (0.0–1.5)	224	Reference
Quartile 2 (1.6–2.4)	145	0.1 (−0.3, 0.5)	Quartile 2 (1.6–2.4)	193	−0.4 (− 0.9, 0.1)
Quartile 3 (2.5–3.1)	193	0.3 (−0.1, 0.6)	Quartile 3 (2.5–3.1)	200	0.0 (−0.5, 0.5)
Quartile 4 (3.2–13)	174	0.1 (−0.3, 0.6)	Quartile 4 (3.2–13)	206	0.0 (−0.6, 0.6)
P for trend		0.45	P for trend		0.62
P for interaction		continuous = 0.48, quartiles = 0.34			
*Ponderal index (kg/m* ^*3*^ *)* ^b^					
Pre-pregnancy sedentary time (hours/day)			Pre-pregnancy sedentary time (hours/day)		
Continuous (hrs)	691	0.2 (− 0.2, 0.6)	Continuous (hrs)	733	0.0 (−0.3, 0.3)
Quartile 1 (0.0–1.4)	139	Reference	Quartile 1 (0.0–1.4)	185	Reference
Quartile 2 (1.5–2.0)	184	0.8 (−0.6, 2.3)	Quartile 2 (1.5–2.0)	222	0.4 (−1.0, 1.8)
Quartile 3 (2.1–2.9)	104	0.3 (−0.8, 1.4)	Quartile 3 (2.1–2.9)	115	0.7 (−1.2, 2.7)
Quartile 4 (3.0–13)	204	1.0 (−0.03, 2.1)	Quartile 4 (3.0–13)	211	0.3 (−1.0, 1.6)
P for trend		0.17	P for trend		0.64
P for interaction		continuous = 0.45, quartiles = 0.70			
Early pregnancy sedentary time (hours/day)			Early pregnancy sedentary time (hours/day)		
Continuous (hrs)	700	0.2 (−0.2, 0.6)	Continuous (hrs)	825	−0.1 (− 0.6, 0.4)
Quartile 1 (0.0–1.5)	190	Reference	Quartile 1 (0.0–1.5)	224	Reference
Quartile 2 (1.6–2.4)	144	−0.2 (−1.3, 0.9)	Quartile 2 (1.6–2.4)	195	−2.5 (−5.3, 0.4)
Quartile 3 (2.5–3.1)	192	0.2 (−0.9, 1.3)	Quartile 3 (2.5–3.1)	200	−1.5 (−4.5, 1.4)
Quartile 4 (3.2–13)	174	0.2 (−1.4, 1.8)	Quartile 4 (3.2–13)	206	−1.5 (− 4.3, 1.3)
P for trend		0.74	P for trend		0.38
P for interaction		continuous = 0.35, quartiles = 0.55			

^a^Model is adjusted for maternal age (years), white race, nulliparity, pre-pregnancy BMI category (underweight/normal weight/overweight/obese), high school education or more, marital status (married/single), smoking during pregnancy, alcohol use during pregnancy, gestational age at delivery (weeks), and pre-pregnancy moderate/vigorous leisure time physical activity duration (quartiles) for pre-pregnancy sedentary time analyses or early pregnancy moderate/vigorous leisure time physical activity duration (quartiles) for early pregnancy sedentary time analyses

^b^Results are excluding outliers for ponderal index (> 400 kg/m^3^)

Similarly, there were no statistically significant associations of replacing leisure time sedentary behavior with light physical activity or moderate/vigorous physical activity with mean offspring birthweight (Table 4). Estimates for differences in mean birthweight associated with replacing leisure time sedentary behavior with light physical activity or moderate/vigorous physical activity were similar in women with low or high levels of sedentary time.

**Table 4:** Substitution of light or moderate/vigorous leisure time physical activity for sedentary behavior and associations with offspring birthweight (g)

	Overall	Low sedentary time	High sedentary time
	Mean difference^a^ (95% CI)		
Pre-pregnancy (n = 1373)			
Light physical activity (hours/week)	−48 (− 116, 21)	−54 (− 147, 39)	−57 (− 154, 41)
Moderate/vigorous physical activity (hours/week)	0.4 (− 5.2, 6.0)	− 2.7 (− 13, 7.8)	4.5 (− 4.0, 13)
P for interaction = 0.96 for light physical activity, 0.28 for moderate/vigorous physical activity			
Early pregnancy (*n* = 1535)			
Light physical activity (hours/week)	−1.2 (− 19, 17)	11 (− 5.5, 27)	−8.3 (− 44, 27)
Moderate/vigorous physical activity (hours/week)	−4.7 (− 12, 2.0)	−10 (− 22, 1.8)	−1.9 (− 12, 8.3)
P for interaction = 0.18 for light physical activity, 0.67 for moderate/vigorous physical activity			

^a^Model is adjusted for pre-pregnancy or early pregnancy sedentary time (hours/week), total pre-pregnancy or early pregnancy leisure time (hours/week), maternal age (years), white race, nulliparity, pre-pregnancy BMI category (underweight/normal weight/overweight/obese), high school education or more, marital status (married/single), smoking during pregnancy, alcohol use during pregnancy, gestational age at delivery (weeks), and offspring sex

## Discussion

In our study, we did not observe associations between pre-pregnancy or early pregnancy leisure time sedentary behavior and mean offspring birth size. Replacing sedentary behavior with light or moderate/vigorous physical activity was also not associated with mean offspring birth size. In addition, we did not observe effect modification of associations by offspring sex.

Our findings are similar to several [19–21], but not all [16–18], reports. Prospective cohort studies of singleton births in the United Kingdom [19], the Netherlands [20], and India [21], with either objectively measured or self-reported sedentary behavior in early, mid, and late pregnancy did not find associations between duration of sedentary time or percent of total time spent in sedentary behavior and mean birthweight or macrosomia. These studies were small, with study sizes ranging from 111 to 546 participants, and may not have been adequately powered to detect smaller, but still clinically important differences in birthweight associated with maternal sedentary behavior. A larger prospective cohort study of singleton births (*N* = 11,759) in the United Kingdom by Hayes et al. found that a sedentary lifestyle in early and mid-pregnancy was associated with 21 g (95% CI: -3.5, − 40) and 22 g (95% CI: -3.7, − 40) lower mean offspring birthweight, respectively, compared to an active lifestyle [16]. In this study by Hayes et al., sedentary behavior was characterized by self-report of ‘mostly sitting’ during the day, and did not quantify duration of sedentary behavior. A case-control study in Brazil reported 30% increased risk for intrauterine growth restriction (OR = 1.30; 95% CI: 1.27, 1.31) associated with sedentary behavior during mid-pregnancy [17], but the increased risk observed may be an overestimate due to absence of control for confounding variables. A cross-sectional study in Ireland reported that women who delivered macrosomic infants spend 2.0 more hours (95% CI: 0.3, 3.7) in sedentary time during late pregnancy compared to women who delivered normal weight infants [18]. In that study, sedentary behavior included sleep, which is also associated with offspring birth size [28] and may explain the observed association between sedentary behavior and macrosomia. Our study addressed limitations of previous studies by controlling for several potential confounders and quantifying leisure time sedentary behavior duration. Additionally, we considered associations of replacing leisure time sedentary behavior with light physical activity or moderate/vigorous physical activity in a substitution model. However, we were not able to reliably estimate associations of maternal sedentary behavior with small- or large-for-gestational age due to small numbers of these outcomes in our study population.

Strengths of our study include its prospective design, multiple measures of birth size, adjustment for variables associated with sedentary behavior in our study population (education, BMI, parity, and marital status), and use of non-parametric calibration weighting to address missing data and increase the power of our study. Despite this approach to missing data, our study may not have been adequately powered to detect small differences in birthweight that have been reported in previous studies. The results of the calibration weighting approach are representative of the full cohort under a missing at random assumption, where availability of sedentary behavior data depends only on the matching variables. This is plausible in our data because the availability of sedentary behavior data is dependent on enrollment period, but we were unable to include lifestyle characteristics, such as physical activity, smoking, or alcohol, in the calculation of adjustment weights due to missingness in these variables in the full cohort, which may have reduced the additional precision we were able to achieve. Omega study participants were generally healthy, with high participation in physical activity, low smoking rates, and other healthy behaviors. Our population may be less sedentary than other populations as the average sedentary time in our population was much lower than in the general population, or adverse effects of sedentary behavior may not be as strong in our population compared to less healthy populations. We only assessed a limited number of leisure time sedentary behaviors (watching TV and quiet activities such as reading and knitting), which may also explain the low average sedentary behavior in our study if participants tended to do other sedentary activities. Limited measurement of sedentary behavior in our study may explain the observed lack of association between maternal sedentary behavior and mean infant birthweight we observed. Sedentary behavior was also recalled and self-reported, which may have introduced measurement error into our study. Validity of self-reported time spent watching television among pregnant women has not been assessed; however, validity has been shown to be good compared to accelerometer data (*r* = 0.83) in non-pregnant adults [29]. Finally, the majority of participants in the Omega study were of high socioeconomic status [22], due to the geographical area and study hospitals from which participants were recruited. Results may not be generalizable to more socioeconomically diverse populations.

## Conclusions

In summary, we did not find associations of pre-pregnancy or early pregnancy sedentary time and mean offspring birth size. Pre-pregnancy and early pregnancy sedentary behavior may have important adverse effects on maternal health, but our results do not support associations of maternal sedentary behavior before or during pregnancy with mean offspring birth size.

## Abbreviations

BMI: Body mass index
CI: Confidence interval
MET: Metabolic equivalent
OR: Odds ratio

## References

[1] Tremblay MS, Aubert S, Barnes JD, Saunders TJ, Carson V, Latimer-Cheung AE, Chastin SFM, Altenburg TM, Chinapaw MJM (2017). Sedentary behavior research network (SBRN) - terminology consensus project process and outcome. Int J Behav Nutr Phys Act.

[2] Biswas A, Oh PI, Faulkner GE, Bajaj RR, Silver MA, Mitchell MS, Alter DA (2015). Sedentary time and its association with risk for disease incidence, mortality, and hospitalization in adults: a systematic review and meta-analysis. Ann Intern Med.

[3] Grontved A, Hu FB (2011). Television viewing and risk of type 2 diabetes, cardiovascular disease, and all-cause mortality: a meta-analysis. Jama.

[4] Young DR, Hivert MF, Alhassan S, Camhi SM, Ferguson JF, Katzmarzyk PT, Lewis CE, Owen N, Perry CK, Siddique J (2016). Sedentary behavior and cardiovascular morbidity and mortality: a science advisory from the American Heart Association. Circulation.

[5] Garber CE, Blissmer B, Deschenes MR, Franklin BA, Lamonte MJ, Lee IM, Nieman DC, Swain DP (2011). American College of Sports Medicine position stand. Quantity and quality of exercise for developing and maintaining cardiorespiratory, musculoskeletal, and neuromotor fitness in apparently healthy adults: guidance for prescribing exercise. Med Sci Sports Exerc.

[6] Di Fabio DR, Blomme CK, Smith KM, Welk GJ, Campbell CG (2015). Adherence to physical activity guidelines in mid-pregnancy does not reduce sedentary time: an observational study. Int J Behav Nutr Phys Act.

[7] Matthews CE, Chen KY, Freedson PS, Buchowski MS, Beech BM, Pate RR, Troiano RP (2008). Amount of time spent in sedentary behaviors in the United States, 2003-2004. Am J Epidemiol.

[8] Zhang C, Solomon CG, Manson JE, Hu FB (2006). A prospective study of pregravid physical activity and sedentary behaviors in relation to the risk for gestational diabetes mellitus. Arch Intern Med.

[9] Alberico S, Montico M, Barresi V, Monasta L, Businelli C, Soini V, Erenbourg A, Ronfani L, Maso G (2014). The role of gestational diabetes, pre-pregnancy body mass index and gestational weight gain on the risk of newborn macrosomia: results from a prospective multicentre study. BMC Pregnancy Childbirth.

[10] Healy GN, Dunstan DW, Salmon J, Shaw JE, Zimmet PZ, Owen N (2008). Television time and continuous metabolic risk in physically active adults. Med Sci Sports Exerc.

[11] Fowden AL, Ward JW, Forhead AJ, Gluckman P, Hanson M (2006). Control of fetal metabolism: relevance to developmental origins of health and disease. Developmental Origins of Health and Disease.

[12] Eriksson J, Forsen T, Tuomilehto J, Osmond C, Barker D (2001). Size at birth, childhood growth and obesity in adult life. Int J Obes Relat Metab Disord.

[13] Santos MS, Joles JA (2012). Early determinants of cardiovascular disease. Best Pract Res Clin Endocrinol Metab.

[14] Oken E, Gillman MW (2003). Fetal origins of obesity. Obes Res.

[15] Gampel SB, Nomura Y (2014). Short and long-term effects of compromised birth weight, head circumference, and Apgar scores on neuropsychological development. Jof Psychol Abnorm Child.

[16] Both MI, Overvest MA, Wildhagen MF, Golding J, Wildschut HI (2010). The association of daily physical activity and birth outcome: a population-based cohort study. Eur J Epidemiol.

[17] Takito MY, Benicio MH (2010). Physical activity during pregnancy and fetal outcomes: a case-control study. Revista de Saude Publica.

[18] Reid EW, McNeill JA, Alderdice FA, Tully MA, Holmes VA (2014). Physical activity, sedentary behaviour and fetal macrosomia in uncomplicated pregnancies: a prospective cohort study. Midwifery.

[19] Hayes L, Bell R, Robson S, Poston L (2014). Association between physical activity in obese pregnant women and pregnancy outcomes: the UPBEAT pilot study. Ann Nutr Metab.

[20] Ruifrok AE, Althuizen E, Oostdam N, van Mechelen W, Mol BW, de Groot CJ, van Poppel MN (2014). The relationship of objectively measured physical activity and sedentary behaviour with gestational weight gain and birth weight. J Pregnancy.

[21] Dwarkanath P, Muthayya S, Vaz M, Thomas T, Mhaskar A, Mhaskar R, Thomas A, Bhat S, Kurpad A (2007). The relationship between maternal physical activity during pregnancy and birth weight. Asia Pac J Clin Nutr.

[22] Rudra CB, Sorensen TK, Luthy DA, Williams MA (2008). A prospective analysis of recreational physical activity and preeclampsia risk. Med Sci Sports Exerc.

[23] Chan KCG, Yam SCP, Zhang Z (2016). Globally efficient non-parametric inference of average treatment effects by empirical balancing calibration weighting. J Royal Stat Soc Series B Stat Methodol.

[24] Breslow NE, Lumley T, Ballantyne CM, Chambless LE, Kulich M (2009). Using the whole cohort in the analysis of case-cohort data. Am J Epidemiol.

[25] Faerch K, Lau C, Tetens I, Pedersen OB, Jorgensen T, Borch-Johnsen K, Glumer C (2005). A statistical approach based on substitution of macronutrients provides additional information to models analyzing single dietary factors in relation to type 2 diabetes in danish adults: the Inter99 study. J Nutr.

[26] 2008 Physical Activity Guidelines for Americans [www.health.gov/paguidelines]. Accessed 18 July 2017.

[27] R Development Core Team. R: a language and environment for statistical computing. Vienna: R Foundation for Statistical Computing; 2013.

[28] Zafarghandi N, Hadavand S, Davati A, Mohseni SM, Kimiaiimoghadam F, Torkestani F (2012). The effects of sleep quality and duration in late pregnancy on labor and fetal outcome. J Matern Fetal Neonatal Med.

[29] Matton L, Wijndaele K, Duvigneaud N, Duquet W, Philippaerts R, Thomis M, Lefevre J (2007). Reliability and validity of the Flemish physical activity computerized questionnaire in adults. Res Q Exerc Sport.

